# Human–Exoskeleton Coupling Simulation for Lifting Tasks with Shoulder, Spine, and Knee-Joint Powered Exoskeletons

**DOI:** 10.3390/biomimetics9080454

**Published:** 2024-07-25

**Authors:** Asif Arefeen, Ting Xia, Yujiang Xiang

**Affiliations:** 1 School of Mechanical and Aerospace Engineering, Oklahoma State University, Stillwater, OK 74078, USA; asif.arefeen@okstate.edu; 2 Department of Mechanical Engineering, Northern Illinois University, DeKalb, IL 60115, USA; txia@niu.edu

**Keywords:** wearable robot, human–exoskeleton coupling, powered exoskeletons, optimal control, gradient-based optimization

## Abstract

In this study, we introduce a two-dimensional (2D) human skeletal model coupled with knee, spine, and shoulder exoskeletons. The primary purpose of this model is to predict the optimal lifting motion and provide torque support from the exoskeleton through the utilization of inverse dynamics optimization. The kinematics and dynamics of the human model are expressed using the Denavit–Hartenberg (DH) representation. The lifting optimization formulation integrates the electromechanical dynamics of the DC motors in the exoskeletons of the knee, spine, and shoulder. The design variables for this study include human joint angle profiles and exoskeleton motor current profiles. The optimization objective is to minimize the squared normalized human joint torques, subject to physical and task-specific lifting constraints. We solve this optimization problem using the gradient-based optimizer SNOPT. Our results include a comparison of predicted human joint angle profiles, joint torque profiles, and ground reaction force (GRF) profiles between lifting tasks with and without exoskeleton assistance. We also explore various combinations of exoskeletons for the knee, spine, and shoulder. By resolving the lifting optimization problems, we designed the optimal torques for the exoskeletons located at the knee, spine, and shoulder. It was found that the support from the exoskeletons substantially lowers the torque levels in human joints. Additionally, we conducted experiments only on the knee exoskeleton. Experimental data indicated that using the knee exoskeleton decreases the muscle activation peaks by 35.00%, 10.03%, 22.12%, 30.14%, 16.77%, and 25.71% for muscles of the erector spinae, latissimus dorsi, vastus medialis, vastus lateralis, rectus femoris, and biceps femoris, respectively.

## 1. Introduction

In recent years, the use of exoskeleton technology has seen growth in areas such as the military, neurotherapy, building sectors, industrial production, and everyday tasks. However, it is believed that close to 8.8 million individuals aged between 21 and 64 in the U.S. face challenges when it comes to lifting items [1]. As a result, the design and control of powered exoskeletons are crucial for tasks involving lifting.

In the past few years, there has been an increased exploration of dynamic modeling techniques rooted in optimization to study the interaction between exoskeletons and their users [2,3,4,5]. The literature mainly focuses on single-joint human–exoskeleton interaction. However, this research focuses on multiple-joint human–exoskeleton interactions and studies their effects on joint torques, human energy consumption, and motion predictions. This is the unique contribution of this work. Millard et al. [6] constructed a 2D 12-degree-of-freedom (DOF) human model with 9-DOF powered spine and hip exoskeletons. They employed a direct control method founded on sequential quadratic programming (SQP) to anticipate human motions and the associated forces during lifting activities, aiming to decrease the risk of injuries to the lower back. Another innovation was the introduction of a 1-DOF back exoskeleton powered by pneumatic drive to aid in lifting, as mentioned in [7]. Observations indicated that this newly developed model could reduce muscle activity during lifting, leading to a decrease in lower back discomfort by approximately 18% to 25%. Several other powered exoskeletons designed for the upper limbs have been introduced to support workers in tasks like overhead lifting or manual work [8,9,10]. Arefeen and Xiang [11] proposed a physics-based optimization formulation to determine the best control for a powered elbow exoskeleton, enhancing collaboration between humans and robots in lifting tasks. Furthermore, there has been a surge in the development of upper-limb powered exoskeletons aimed at assisting the elderly and those in rehabilitation. For instance, Kiguchi et al. [12] designed a novel 7-DOF powered exoskeleton for the upper limbs, taking into account the natural shoulder movement observed in experiments. Rahman et al. [13] offered a model and control strategy for a 2D exoskeleton powered by a DC motor for the shoulder and elbow, which was designed to support rehabilitation and everyday tasks.

Numerous studies have primarily concentrated on developing and controlling exoskeletons for the knee joint, with the aim of decreasing metabolic expenditure, mitigating muscle tiredness, and reducing muscle activity during squat-lifting tasks [14,15,16,17,18,19]. Furthermore, various exoskeletons have been engineered to support movements similar to squat lifting, such as standing up and sitting [20,21,22]. Recent investigations [23,24,25] have highlighted significant advancements in the domain of motorized knee and lower-extremity exoskeletons, particularly in their structural and actuation features. Moreover, there is a growing interest in adopting learning-driven control techniques among scholars [26,27,28,29,30,31,32,33,34], all of which enhance the effectiveness of these tools in facilitating squat lifting, ambulation, and recovery processes.

This research introduces a multi-joint powered exoskeleton optimal control strategy, encompassing the knee, spine, and shoulder. Different couplings of exoskeletons include (1) spine and shoulder exoskeletons; (2) spine, shoulder, and knee exoskeletons; and (3) knee exoskeletons for squat-lifting tasks. We showcase the coupled knee, spine, and shoulder exoskeleton torque profiles resulting from optimization. The focus of this research is multi-joint exoskeleton collaborations for lifting tasks using optimization and the effects of different exoskeleton combinations. The proposed work uses inverse dynamics optimization to predict the human–exoskeleton symmetric lifting motion and optimal assistive torques for the multiple joints. An SQP algorithm in the sparse nonlinear optimizer (SNOPT) is used to solve the lifting optimization problem [35,36]. Simulation comparisons are carried out for different couplings of exoskeletons between the cases with and without exoskeletons. In addition, the predicted knee exoskeleton’s optimal results are used in the previously developed human–exoskeleton lifting control strategy. Finally, experimental validations are presented, focusing solely on the knee exoskeleton.

The contents are organized as follows: Section 2 first describes subject-specific coupled human–exoskeleton models and details the human–exoskeleton coupled equations of motion (EOMs). Section 3 presents the lifting optimization formulation. Section 4 discusses the brief exoskeleton control strategy and experimental setup. Simulation results and lifting validations with knee exoskeletons are presented in Section 5. Discussion and concluding remarks are provided in Section 6 and Section 7, respectively.

## 2. Methods

### 2.1. Subject-Specific Coupled Human–Exoskeleton Model

**Figure 1:**
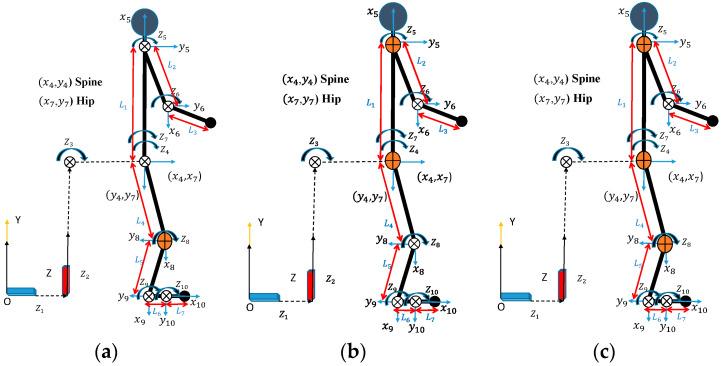
The 2D human skeletal model with 1-DOF powered exoskeletons attached at the (**a**) knee joint (orange), (**b**) spine and shoulder joints (orange), and (**c**) knee, spine, and shoulder joints (orange).

This work focuses on the utilization of a 2D 10-DOF human skeletal model [37] coupled with various combinations of 1-DOF powered knee, spine, and shoulder exoskeletons, as depicted in Figure 1. The sagittal plane serves as the axis of symmetry for the model. To simulate the system, the mass and inertia characteristics of the exoskeleton are taken into account through mathematical analysis. The proposed 2D model has symmetry. This means that the left and right limbs have the same mass and inertia. In addition, the two legs and two arms are combined into one single limb for a 2D model. The single limb has calculated mass, inertia, and strength considering both legs and arms. The mass and inertia of the exoskeleton are added to the upper and lower limbs, respectively. The construction of the human model employs the Denavit–Hartenberg (DH) method [38]. Every DOF relates to the rotation or translation between two parts of the body connected by either a rotating or sliding connection. The degree of freedom for both rotational and translational joints is measured along the local z-direction. In the global Y-Z plane, all local rotation joints ($z_{3}\sim z_{10}$) rotate in a clockwise direction. The Xsens motion capture system provides the subject’s body measurement data. The human model’s DH parameters are shown in Table 1, with L1 to L7 representing the lengths of the respective joint links in the human body. These joint link lengths are different and unique for different humans. As a result, our 2D model can be scaled for different individuals.

### 2.2. Human–Exoskeleton Coupled Equations of Motion (EOMs)

The human model’s kinematics and dynamics are represented using recursive kinematics and Lagrangian dynamics. The human dynamics equation can be written as follows [39,40,41]:

(1) $$\tau_{h_{i}}=tr\left(\frac{\partial A_{i}}{\partial q_{i}}D_{i}\right)-g^{T}\frac{\partial A_{i}}{\partial q_{i}}E_{i}-f_{k}^{T}\frac{\partial A_{i}}{\partial q_{i}}F_{i}-G_{i}^{T}A_{i-1}z_{0}$$

where $\tau_{h_{i}}$ is the human torque at the *ith* joint. On the right-hand side of Equation (1), the first term is inertia and Coriolis torque, the second term is the torque due to gravity, the third term is the torque due to external forces, and the fourth term is the torque due to external moments. **A** is the global position matrix, **D** is the backward recursive inertia and Coriolis matrix, **E** is the backward recursive gravity vector, **F** is the backward recursive external force vector, and **G** is the backward recursive external moment vector [41].

The optimization-based prediction of dynamic human lifting movements incorporates models of the electromechanical behaviors of DC motors found in exoskeletons. The dynamics equations can be expressed as follows [42]:

(2) $$L\frac{dI}{dt}=V-K\frac{d\theta }{dt}-RI$$

(3) $$T_{motor}=KI$$

(4) $$T_{l}=T_{motor}-J_{m}\frac{d^{2}\theta }{dt^{2}}-b\frac{d\theta }{dt}$$

where *V*, I, L, and R are the voltage input, current, inductance, and resistance, respectively. The mechanical terms $J_{m}$, b, K, and θ are the rotor moment of inertia, coefficient of viscous friction of the motor, motor torque constant, and rotor angle, respectively. $T_{motor}$ is the motor output torque, and $T_{l}$ is the load torque of the exoskeleton. In this study, the current I is parameterized using B-splines as design variables. Then, the motor torque and exoskeleton load torque are calculated from Equations (3) and (4) directly. In addition, the sensitivity of the exoskeleton torque with respect to the current is derived as in [19]. The gearbox ratios (${GB}_{r}$) are chosen so that the devices can provide the required torque output. Here, the exoskeleton includes the motor and the gearbox, so the output torque ($\tau_{e})$ of the exoskeleton can be expressed as follows:

(5) $$\tau_{e}={GB}_{r}\times T_{l}$$

The coupled human–exoskeleton EOMs and sensitivity analysis are formed using a recursive Lagrangian dynamics formulation. The overall dynamics can be expressed as follows:

(6) $$\tau_{h_{i}}+\tau_{e_{i}}=tr\left(\frac{\partial A_{i}}{\partial q_{i}}D_{i}\right)-g^{T}\frac{\partial A_{i}}{\partial q_{i}}E_{i}-f_{k}^{T}\frac{\partial A_{i}}{\partial q_{i}}F_{i}-G_{i}^{T}A_{i-1}z_{0}$$

where $\tau_{e_{i}}$ is the exoskeleton output torque for the *ith* joint. The detailed explanations of the zero-moment point (ZMP) and ground reaction forces (GRFs) are calculated from Equation (1) and are available in the literature [39,40].

## 3. Lifting Optimization Formulation

### 3.1. Design Variables

The interpolation of the exoskeletons’ currents I(t) and human joint angle profiles q(t) is achieved using cubic B-splines [40]. The design variables (x) consist of the control points $P_{human}$ related to the human joint angle and the control points $P_{current}$ associated with the exoskeleton current. Hence, the formulation of design variables can be expressed as $x={\left[\begin{array}{cc} P_{human}^{T} & P_{current}^{T} \end{array}\right]}^{T}$.

### 3.2. Objective Functions

This work considers the total squared normalized torques of human joints as the objective function [19].

(7) $$\underset{x}{\min}J\left(x\right)=\sum_{i=3}^{n}\int_{0}^{T}{\left[\frac{\tau_{hi}(x)}{{(\tau }_{i}^{U}-\tau_{i}^{L})}\right]}^{2}dt$$

where T is the specified total time for the lifting task, while $\tau_{i}^{U}$ and $\tau_{i}^{L}$ are the upper and lower human torque limits for the *ith* joint, respectively.

### 3.3. Constraints

The physical and task-based constraints are (1) joint angle limits, (2) joint torque limits, (3) feet contact position, (4) hand forward position, (5) dynamic stability, (6) collision avoidance, (7) exoskeleton torque limits, (8) initial and final box locations, (9) static conditions at the beginning and end of the motion, and (10) initial, middle, and final joint angles of the knee, ankle, shoulder, spine, hip, and elbow. For time-dependent constraints, constraints (1)–(6) are imposed for humans, and constraint (7) is only for the exoskeletons. Constraints (8)–(10) are the time-independent. The physical joint angle limits for humans $q\left(x,t\right)$ and the joint torque limits for humans $\tau \left(x,t\right)$ and exoskeletons $\tau_{e}\left(x,t\right)$ are depicted in Table 2. Using B-spline interpolation, we determine the human joint angles. The human and exoskeleton joint torque profiles are calculated inversely from the coupled human–exoskeleton EOMs based on state variables. Additionally, global point locations are determined using DH kinematics.

## 4. Exoskeleton Control Strategy and Experiments

### 4.1. Knee Exoskeleton Control Strategy

**Figure 2:**
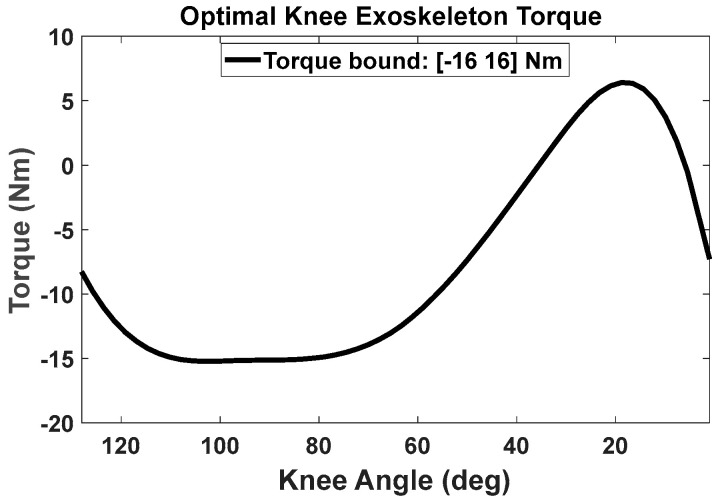
The optimal knee exoskeleton torque as a function of human knee angles during lifting (the knee-joint angle starts from a squat position).

We first employed inverse dynamics optimization formulation to determine the optimal exoskeleton torque in the time domain. Subsequently, we used B-spline interpolation to express the optimal exoskeleton torque as a continuous function in the encoder angle domain for control. To find the exoskeleton torque in the joint angle domain, we carried out a least-squares optimization to minimize the summation of the squared errors between the optimal exoskeleton torque (from inverse dynamics optimization in the time domain) and the exoskeleton torque derived from the encoder angle (via B-spline interpolation in the joint angle domain) [19]. During the experiments, the knee exoskeleton was able to provide assistive torque to the knee joint from the B-spline interpolation function in real time, based on the knee encoder angle. Figure 2 showcases the optimal real-time exoskeleton torque in relation to knee-joint angles, with the maximum assistive torque recorded at 16 Nm for the subject participating in the experiment.

### 4.2. Experimental Procedure

The experiments on the optimal torque control of a powered knee exoskeleton for lifting tasks received Institutional Review Board approval (IRB-21-501). A 27-year-old individual in good health, with no prior injuries, participated after providing written informed consent. The participant measured 1.7 m in height and weighed approximately 68.75 kg.

**Figure 3:**
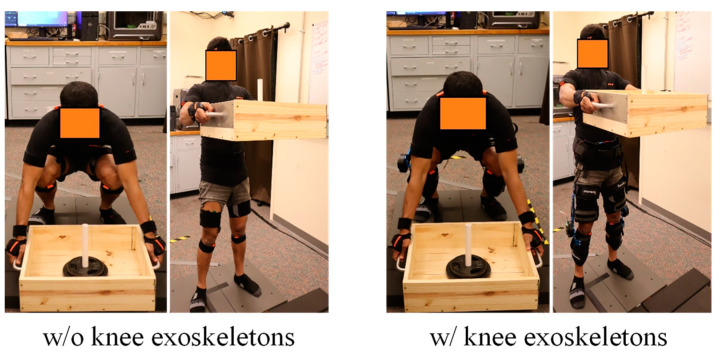
Lifting experiment setup without and with knee exoskeletons.

In this study, the participant attended the lab on two separate occasions. During the initial visit, we recorded the participant’s body measurements. We utilized the Xsens system to capture 3D motion details at a frequency of 60 Hz, adhering to the full-body sensor guidelines (as shown in Figure 3). The study involved monitoring the EMG responses of six specific muscles (erector spinae, latissimus dorsi, vastus medialis, vastus lateralis, rectus femoris, and biceps femoris) on the right side of the body. These measurements were captured using Delsys Trigno devices at a frequency of 2000 Hz. The participant was asked to execute three maximal contractions, lasting 3 s each, for every muscle. These contractions were separated by a 60 s relaxation period, to produce EMG activity that corresponded to maximum voluntary contractions (MVCs) for six muscles. The subject was given a 10 kg box to lift without any assistive device, positioning both feet on the Bertec force plates. The participant was directed to employ a squatting method to lift. The lifting motion was recorded using the MVN Analyze Pro software from Xsens, while the OptiTrack Motive 3.0 software documented the forces exerted on the ground (GRFs) at a rate of 1000 Hz. The EMGworks Acquisition system captured the unprocessed EMG readings. With a three-minute pause in between each lifting session, the task was performed three times. All of the data were processed in MVN Analyze, Motive 3.0, and MATLAB. For the two force plates, the mean value was calculated for each trial’s processed GRFs. In the end, the three experimental attempts’ averages were evaluated against the predicted results.

For the second visit, a lightweight, highly back-drivable, wearable knee exoskeleton developed by Picasso Intelligence, LLC was used in this study. The proposed optimal control strategies were implemented. A training session was held to help the user become familiar with the exoskeleton’s assistance. Finally, the subject was asked to wear Xsens whole-body sensors, EMG sensors, and the exoskeleton to perform the lifting task again.

## 5. Results

The nonlinear optimization problem of lifting was efficiently solved using the SQP algorithm in SNOPT [35]. The initial guess for the design variables was set to zero, $x_{0}={\left[\begin{array}{cc} P_{human}^{T} & P_{current}^{T} \end{array}\right]}^{T}=[0]$. For cubic B-spline interpolation, we considered five control points (nctrl = 5) for each joint. There was a total of 50 (n*nctrl) design variables and 217 nonlinear constraints for lifting optimization without exoskeletons. The optimal solution was obtained in 1.31 s CPU time. The electrical and mechanical parameters of the DC motor are available in [11]. For the lifting optimization with the exoskeletons, there were (1) 55 (n*nctrl+number of exo joints*nctrl) design variables and 222 nonlinear constraints for the knee exoskeleton, (2) 60 design variables and 227 nonlinear constraints for the coupled spine and shoulder exoskeletons, and (3) 65 design variables and 232 nonlinear constraints for the coupled knee, spine, and shoulder exoskeletons. The exoskeletons’ torque limits were considered in the optimization: [−16, 16] Nm for the knee exoskeleton, [−36, 36] Nm for the spine exoskeleton and [−12, 12] Nm for the shoulder exoskeleton. The knee, spine, and shoulder exoskeletons’ weights were 2.25 kg, 3.4 kg, and 1.2 kg, respectively. The exoskeletons’ weight and torque limits were chosen based on the actual exoskeleton developed by Picasso Intelligence, LLC. The optimal solutions were obtained in 2.43, 10.68, and 13.79 s CPU time, respectively. An Intel^®^ Core™ i7 2.11 GHz CPU and 16 GB RAM computer was used for the optimization. The input data for the box-lifting task are given in Table 3.

**Figure 4:**
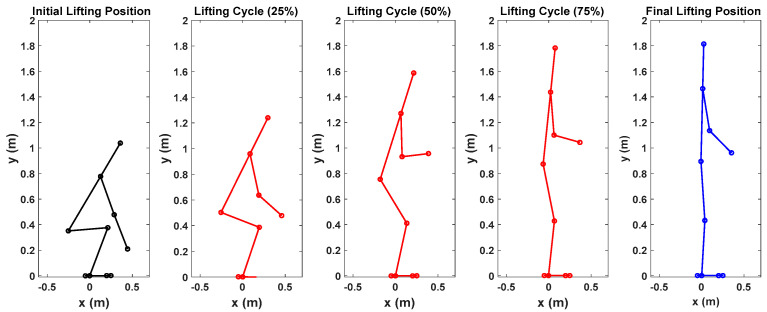
Snapshots of predicted 2D human lifting motion without exoskeleton assistance.

**Figure 5:**
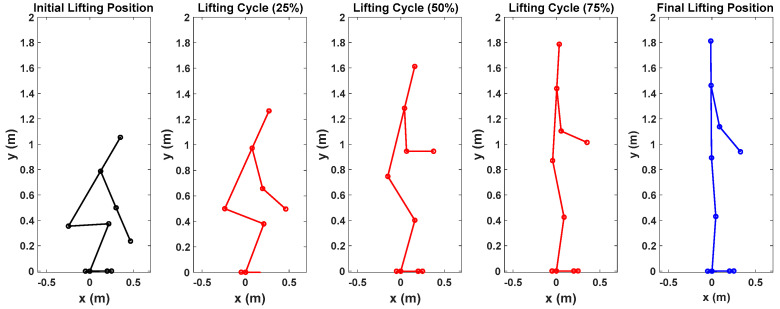
Snapshots of predicted 2D human lifting motion with spine and shoulder exoskeleton assistance.

**Figure 6:**
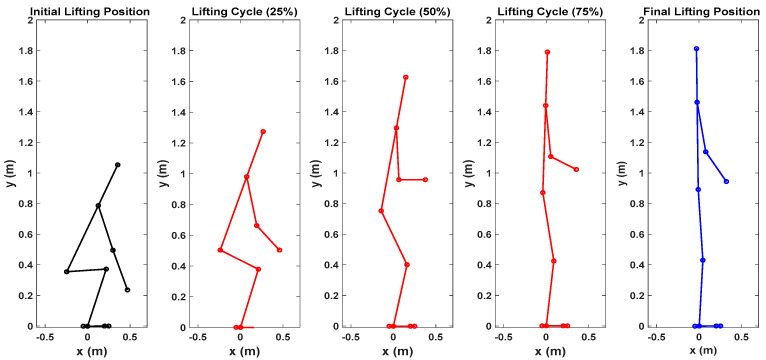
Snapshots of predicted 2D human lifting motion with knee, spine, and shoulder exoskeleton assistance.

**Figure 7:**
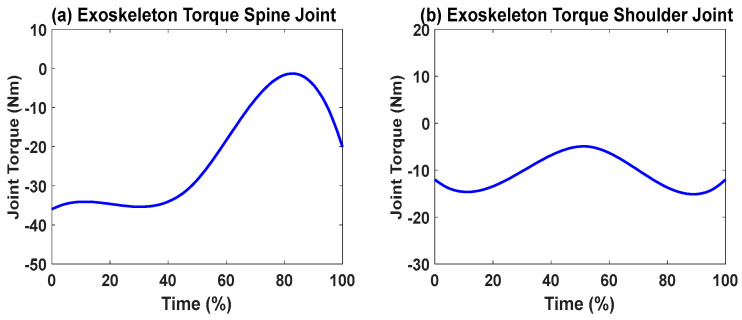
Coupled spine and shoulder exoskeletons’ optimal joint torque profiles from the simulation.

**Figure 8:**
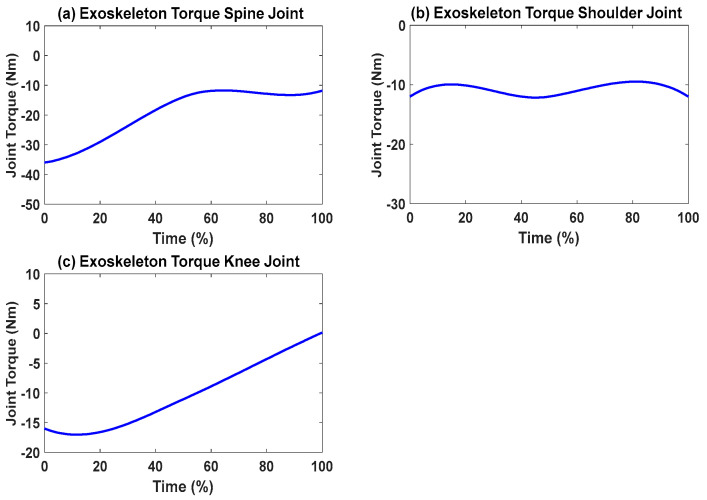
Coupled knee, spine, and shoulder exoskeletons’ optimal joint torque profiles from the simulation.

**Figure 9:**
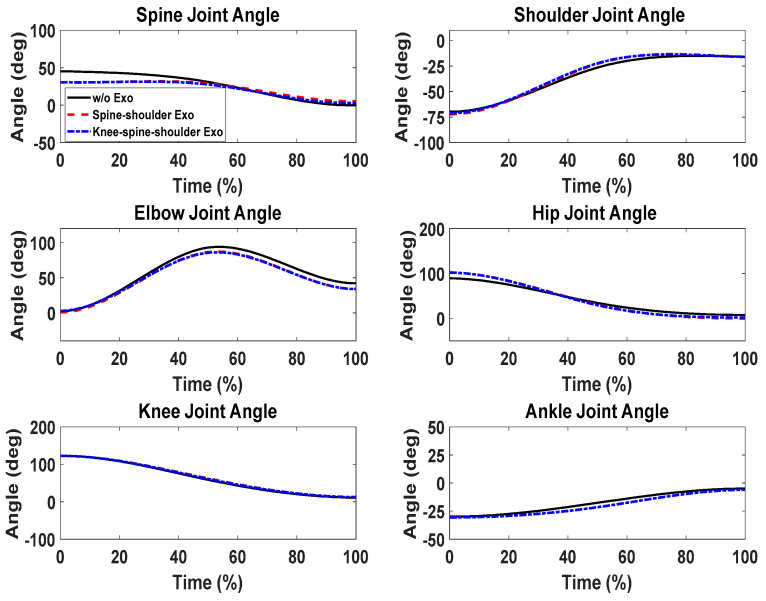
Comparison of predicted human joint angles between the cases with and without exoskeleton assistance.

**Figure 10:**
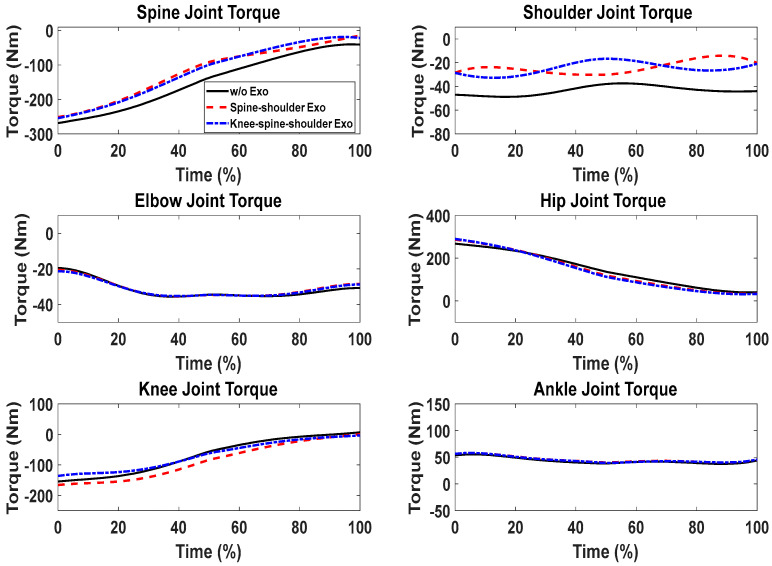
Comparison of predicted human joint torques between the cases with and without exoskeleton assistance.

**Figure 11:**
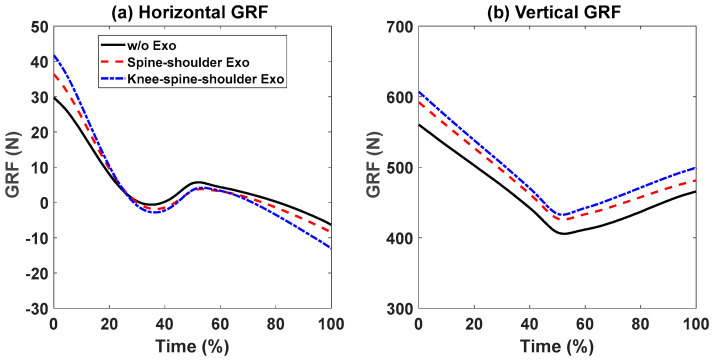
Comparison of predicted human vertical and horizontal GRF profiles between the cases with and without exoskeleton assistance.

First, snapshots of the predicted lifting motion at different lifting times are depicted in Figure 4, Figure 5 and Figure 6. The predicted snapshots of the lifting motions are similar. This was expected, meaning that wearing different exoskeletons has less effect on the predicted motions for the simulation, but there were some differences in the middle postures at 50% of the lifting process. The differences were mainly for the knee and elbow. Figure 7 presents the coupled exoskeleton torque profiles at the spine and shoulder joints. The coupled knee, spine, and shoulder joints’ exoskeleton torques are presented in Figure 8. Figure 9 compares the predicted joint angles between the cases with and without exoskeleton assistance. The comparisons of predicted joint torques between the cases with and without exoskeletons are shown in Figure 10. The horizontal and vertical GRFs are compared for the cases with and without exoskeleton assistance in Figure 11. Finally, a comparison of the peak joint torques and human mechanical energy values for different cases is presented in Table 4.

### Lifting Validation with the Knee Exoskeletons

**Figure 12:**
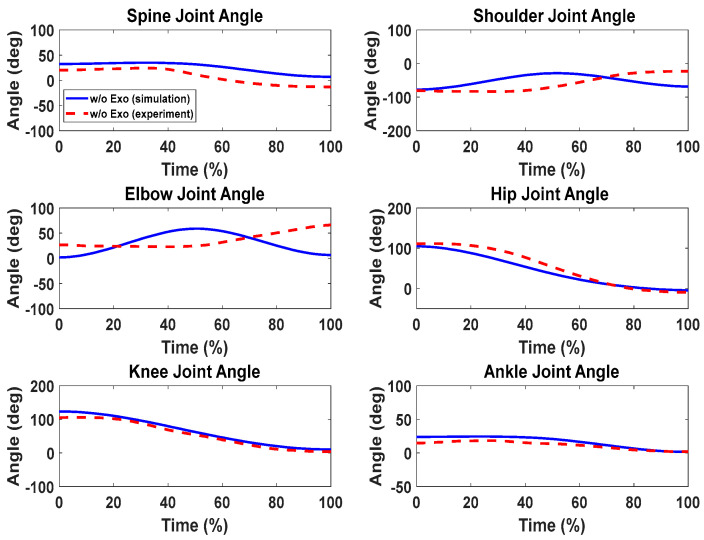
Comparison of human joint angles between simulations and experiments without exoskeleton assistance.

**Figure 13:**
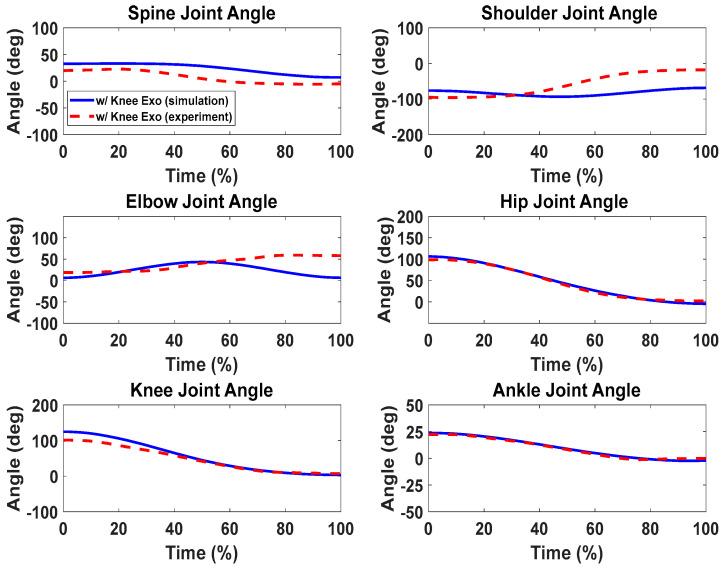
Comparison of human joint angles between simulations and experiments with knee exoskeleton assistance.

**Figure 14:**
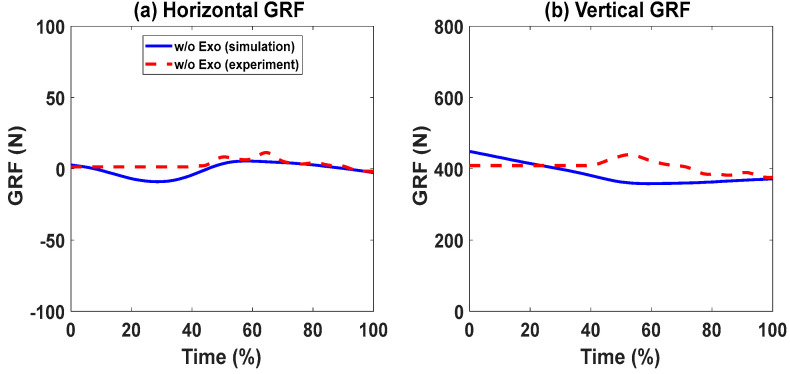
Comparison of GRFs without exoskeletons.

**Figure 15:**
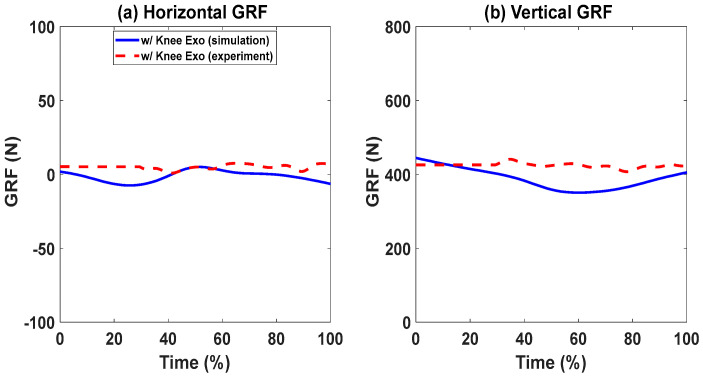
Comparison of GRFs with knee exoskeletons.

**Figure 16:**
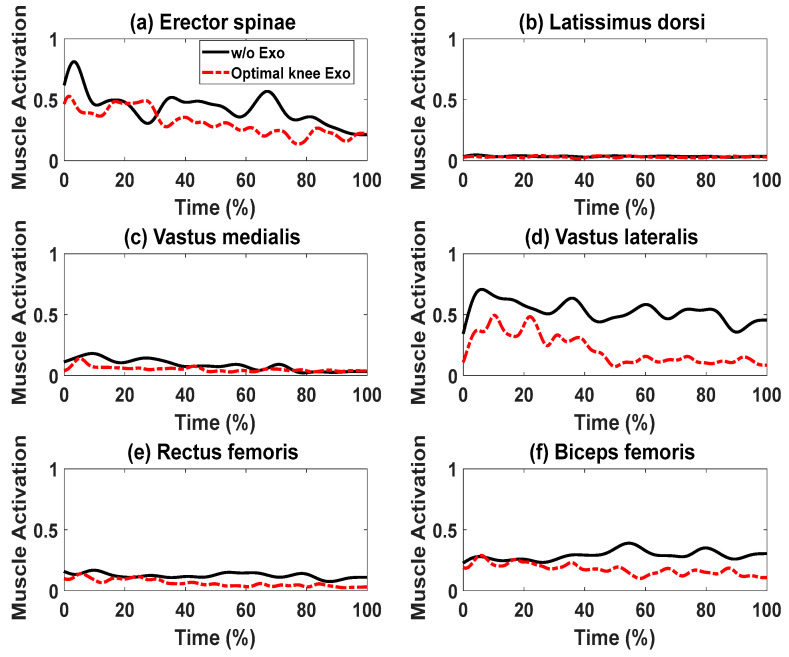
Comparison of muscle activations with and without exoskeletons.

For the cases with and without knee exoskeletons, the comparisons of experimental and simulation joint angles for the human spine, shoulder, elbow, hip, knee, and ankle joints are presented in Figure 12 and Figure 13, respectively. Similarly, Figure 14 and Figure 15 compare the horizontal and vertical GRFs between the experimental and simulation data. Finally, the comparisons of muscle activations are presented in Figure 16.

## 6. Discussion

The proposed optimization formulation for lifting successfully predicted the natural lifting motion across various cycles, both with and without exoskeletons, as illustrated in Figure 4, Figure 5 and Figure 6. Simulations were performed on two different multi-joint exoskeleton couplings. The optimizations identified two sets of optimal exoskeleton torque profiles to assist human lifting motion, as depicted in Figure 7 and Figure 8.

For joint angle comparisons (Figure 9), the predicted joint angle profiles (without exoskeletons) show trends and magnitudes similar to those of the predicted joint angles with exoskeleton assistance. We also noticed a slight discrepancy in the spine joints for the initial 45% of the lifting cycle, indicating a minor change in the lifting strategy for cases with assistance.

We can observe the differences in joint torques between cases without exoskeletons and those with exoskeletons, as illustrated in Figure 10. However, for the cases with coupled spine and shoulder exoskeletons, only the human spine, shoulder, and knee joint torques exhibit different values. Different assistive torques are applied to the spine and shoulder joints. It was observed that the peak values of the human spine and shoulder-joint torque magnitudes decreased by 6.40% and 38.01%, respectively, due to the exoskeleton assistance (Table 4). On the other hand, the human knee-joint torque slightly increased because of the additional weight of the exoskeletons. For other joints, the human torque patterns and magnitudes remained very similar, as no exoskeletons were applied to them. Lastly, for the case with coupled knee, spine, and shoulder exoskeletons, the peak value of the knee-joint torque magnitude decreased by 11.93% compared to the scenario without the knee exoskeleton. In addition, the human mechanical energy for the coupled knee, spine, and shoulder exoskeletons was minimal compared to the other two cases, as presented in Table 4. The spine–shoulder exoskeleton coupling case had slightly larger mechanical energy than the case without exoskeletons, which may have been due to the extra weight of the exoskeletons.

In Figure 11, the horizontal GRFs show similar trends and magnitudes for both cases, with and without exoskeletons. For the vertical GRFs, the trends during lifting are comparable for both cases. However, the vertical GRFs with assistance are higher than those without assistance. The peak value differences are 5.34% and 7.66% for the cases with and without assistance, respectively. These variations in the vertical GRFs can be attributed to the weight of the exoskeletons. The additional mass of the exoskeletons was added to the upper and lower limbs. Therefore, the GRF and joint torques were increased. This is unavoidable when wearing exoskeletons. Lighter exoskeletons using different materials should mitigate this effect. This is also the drawback of wearing exoskeletons, although they can provide extra support for humans.

For the lifting validation with knee exoskeletons, the joint angle comparisons (Figure 12 and Figure 13) show similar trends and magnitudes in the predicted joint angle profiles for both subjects, as compared to the experimental data. However, there are some deviations for the spine, elbow, and shoulder joint angle profiles, which may be due to fewer constraints being imposed on the upper body compared to the lower body. For example, the leg joint angle profiles have good predictions because the feet are fixed on the ground during lifting.

In Figure 14 and Figure 15, the predicted horizontal GRFs exhibit similar trends and magnitude compared to the experimental data for cases with and without exoskeletons. The predicted vertical GRFs also match the experimental GRFs by 5.89% and 8.59%, respectively. The peak value differences are 2.16% and 0.85%, respectively.

In this study, we examined muscle activation from four muscles in the leg, specifically the knee extensors (namely, the rectus femoris, vastus medialis, and vastus lateralis) and the knee flexors (represented by the biceps femoris). Additionally, we included two back muscles, the erector spinae and latissimus dorsi, from the upper body, as depicted in Figure 16. When we compared muscle activation with and without exoskeleton assistance, we noticed similarities in activation patterns in both conditions. However, the use of exoskeletons significantly reduced muscle activations. For instance, the peak activation levels for the erector spinae, latissimus dorsi, vastus medialis, vastus lateralis, rectus femoris, and biceps femoris muscles dropped by 35.00%, 10.03%, 22.12%, 30.14%, 16.77%, and 25.71%, respectively.

The 2D model is used to simulate symmetric lifting. In contrast, the 3D model is able to simulate asymmetric lifting. For symmetric lifting, the 2D model is computationally efficient and has comparable accuracy to the 3D simulation. The differences between the 2D and 3D models for symmetric lifting were discussed in our previous research paper [44]. The SNOPT solver was utilized in this study, which uses a sequential quadratic programming (SQP) algorithm for solving the optimization program. The SQP method uses cost and constraint functions’ gradients (sensitivities), which make the computation fast. Therefore, our method is computationally efficient compared to non-gradient solvers such as genetic algorithms and particle swarm optimization. The drawback of the SQP is that it only finds a local optimum. Therefore, different starting points have to be tested, and the minimum cost function value will be determined as the optimal solution.

For human modeling, the general joint angle limits and torque limits were obtained from the literature [40,43]. The exoskeleton parameters were obtained from the manufacturer’s manual directly. It is true that these physical ranges are not subject-specific, and they are general bounds with approximations. It is tedious to obtain subject-specific strength data, which must be tested using a dynamometer machine. Therefore, theoretically, it is possible that the optimization overfitted the experimental data, especially when the motion or strength was close to the boundaries, but this rarely happens for a lifting task with regular weight.

The optimization formulation achieved system stability through the satisfaction of the zero-moment point (ZMP) constraint. The ZMP constraint was satisfied during the optimization process. This constraint keeps the ZMP within the foot support polygon during the entire lifting process. This constraint guarantees stability during posture transitions in lifting. There are some limitations on the experiments conducted in this study. Only one subject with a knee exoskeleton was validated with the simulation. This is a preliminary validation. Coupled exoskeletons and more subjects will be pursued in future.

## 7. Conclusions

In this work, we used inverse dynamics optimization to predict the coupled multi-joint human exoskeletons’ symmetric lifting motion and optimal exoskeleton torques. The proposed method is subject-specific and has the ability to reduce human multi-joint torques, as well as muscle activations. Overall, the method worked well for the optimal control of powered exoskeletons’ coupling. As a result, dynamic effort and injuries can be mitigated by using the proposed method for lifting tasks. For future work, we will develop a 3D human–exoskeleton coupled model, investigate different lifting strategies, and validate the method against the experimental results.

## Figures and Tables

**Table 1:** DH parameters for the 2D skeletal model.

DOF	ϴ	d	a	α	Translation/Rotation	Branch
1	π	0	0	π/2	Global translation (GT1)	Global branch
2	π/2	L4 + L5	0	−π/2	Global translation (GT2)	
3	0	0	0	0	Global rotation (GR1)	
4	−π/2	0	L1	0	Spine joint rotation (Q1)	Upper body branch
5	π	0	L2	0	Arm joint rotation (Q2)	
6	0	0	L3	0	Elbow joint rotation (Q3)	
7	π/2	0	L4	0	Hip joint rotation (Q4)	Lower body branch
8	0	0	L5	0	Knee joint rotation (Q5)	
9	−π/2	0	L6	0	Ankle joint rotation (Q6)	
10	0	0	L7	0	Subtalar joint rotation (Q7)	

**Table 2:** Constraints.

Time-Dependent	Time-Independent
1. Human joint angle limits [40] $q^{L}\leq q\left(x,t\right)\leq q^{U}$	1. Initial and final box location $p_{human_{hand}}\left(x,t\right)=p_{human_{hand}}^{s}\left(t\right);t=0,T$
2. Human joint torque limits [43] $\tau^{L}\leq \tau \left(x,t\right)\leq \tau^{U}$	2. Initial and final static conditions ${\dot{q}}_{human}\left(x,t\right)=0;\;\;t=0,T$
3. Human feet contact position $p_{feet}\left(x,t\right)=p_{feet}^{s}$	
4. Hand forward position $Z_{wrist}(x,t)-Z_{pelvis}(x,t)\geq 0$	
5. Collision avoidance $d_{human}\left(x,t\right)\geq r_{human}$	3. Initial, middle, and final joint angles $\left|q_{i_{human}}\left(x,t\right)-q_{i_{human}}^{E}\left(t\right)\right|\leq \varepsilon ;$ $t=0,\frac{T}{2},T$ where ε=0.2, and $q_{i\_human}^{E}$ is the experimental joint angle for the human joints.
6. Stability condition $p_{human\_ZMP}\left(x,t\right)\in FSR$	
7. Exoskeleton torque limits $\tau_{e}^{L}\leq \tau_{e}\left(x,t\right)\leq \tau_{e}^{U}$	

The superscripts L and U denote the lower and upper bound of the limit, respectively. $p_{feet}^{s}$ is the specified feet contact position on level ground, $Z_{wrist}$ and $Z_{pelvis}$ are the global Z coordinates of the human wrist and pelvis points, respectively, $d_{human}$ is the calculated distance between the hand and the circle center on the body segment representing the body’s thickness, and $r_{human}$ is the radius of the circle filled on human limbs. The zero-moment point (ZMP) position is inside the foot support region (FSR) for humans [39].

**Table 3:** Parameters for the box-lifting task.

Parameters	
Box weight (kg)	10
Box height (m)	0.15
Box depth (m)	0.65
Initial hand position (x, y, z) (m)	(0.0, 0.070, 0.418)
Final hand position (x, y, z) (m)	(0.0, 1.088, 0.417)
T (s)	1.44

**Table 4:** Comparison of peak joint torques and cost function values for different cases.

Cases	Joints	Peak Joint Torques (Nm)	Human Mechanical Energy (J)
Without exoskeletons	Spine	268.60	622.6
	Shoulder	48.78	
	Knee	154.42	
Coupled spine and shoulder exoskeletons	Spine	251.40	637.7
	Shoulder	30.24	
	Knee	165.73	
Coupled knee, spine, and shoulder exoskeletons	Spine	254.35	586.3
	Shoulder	32.65	
	Knee	135.99	

## Data Availability

The original contributions presented in the study are included in the article, further inquiries can be directed to the corresponding author.
